# Effect of Atmospheric Cold Plasma Treatment on the Adhesion and Tribological Properties of Polyamide 66 and Poly(Tetrafluoroethylene)

**DOI:** 10.3390/ma12040658

**Published:** 2019-02-22

**Authors:** Zoltán Károly, Gábor Kalácska, Jacob Sukumaran, Dieter Fauconnier, Ádám Kalácska, Miklós Mohai, Szilvia Klébert

**Affiliations:** 1 Institute of Materials and Environmental Chemistry, Research Centre for Natural Sciences Hungarian Academy of Sciences, Magyar tudósok krt. 2., H-1117 Budapest, Hungary; mohai.miklos@ttk.mta.hu (M.M.); klebert.szilvia@ttk.mta.hu (S.K.); 2 Institute for Mechanical Engineering Technology, Szent István University, Páter Károly u.1, H-2100 Gödöllő, Hungary; Kalacska.Gabor@gek.szie.hu; 3 Ghent University, Laboratory Soete, Technologiepark Zwijnaarde 903, B-9052 Gent, Belgium; JacobPremKumar.Sukumaran@ugent.be (J.S.); dieter.fauconnier@ugent.be (D.F.); adam.kalacska@ugent.be (Á.K.)

**Keywords:** engineering polymers, cold plasma, diffuse coplanar surface barrier discharges (DCSBD), adhesion, tribology

## Abstract

The surfaces of two engineering polymers including polyamide 66 (PA66) and polytetrafluoroethylene (PTFE) were treated by diffuse coplanar surface barrier discharges in atmospheric air. We found that plasma treatment improved the adhesion of PA66 for either polymer/polymer or polymer/steel joints, however, it was selective for the investigated adhesive agents. For PTFE the adhesion was unaltered for plasma treatment regardless the type of used adhesive. Tribological properties were slightly improved for PA66, too. Both the friction coefficient and wear decreased. Significant changes, again, could not be detected for PTFE. The occurred variation in the adhesion and tribology was discussed on the basis of the occurred changes in surface chemistry, wettability and topography of the polymer surface.

## 1. Introduction

The polyamide 66 (PA 66) and poly(tetrafluoroethylene) (PTFE) are rather different polymers in terms of chemical composition and mechanical properties. However, both plastics are widely used engineering polymers due to a number of favorable properties. PA66 is characterized by good machinability, high strength and stiffness, high hardness and dimensional stability, while PTFE displays exceptional resistance to chemicals. Characteristics they have in common include the relatively high thermal stability among plastics as well as low coefficient of friction. Thus, they frequently find application in industrial areas where outstanding tribological properties are crucial even at elevated temperature. Considering that in most applications polymers are being joined with other structural parts by means of some adhesives, adhesion behavior is of great importance, too. Both adhesion and tribological properties are strongly affected by the chemistry and morphology of the surface. Adhesion are promoted by factors such as high surface energy, good wettability and large area of the contacting surfaces [1,2]. However, polymers typically possess poor surface properties including low surface energy and low wettability due to dearth of polar groups on the surface. For this reason, numerous attempts have been made to improve the surface properties of plastics [3,4,5,6,7]. Among them cold plasma treatments have been gaining growing interest as it offers viable and environmentally benign [8] technical solution even in atmospheric pressure at relatively low cost [9,10,11]. Plasma treatment is capable to improve the adhesive properties by inducing chemical, physical and morphological changes on the polymer surface. Ionized species and free radicals prevailing in the plasma remove organic contamination generally present on the surface. The surface becomes oxidized and activated by formation of polar functional moieties such as hydroxyl, carbonyl or carboxylic acid [12,13]. This results in considerable changes in the chemical composition of the topmost layer and as a consequence, in the surface energy of the treated polymer. The direct electrical charging of the polymer surface due to trapping of ions by CH_2_ groups of the polymer may also contribute to the increment of the surface energy [14]. Plasma treatment can also affect the morphology by exerting etching effect to the surface modifying the surface roughness [15]. Even though cold plasma treatment of various plastics such as polypropylene, polyethylene, polyethylene terephthalate, polyether ether ketone, etc. have been investigated [16,17,18], only limited publication is available on some engineering polymers [19,20,21,22]. In the present paper we reported the surface modification of PA66 and PTFE by diffuse coplanar surface barrier discharges (DCSBD) in atmospheric pressure. We studied the changes in the surface chemistry, wettability and morphology as well as their effect on the adhesion and tribological properties.

## 2. Materials and Methods

### 2.1. Materials and Sample Preparation

Pristine polyamide 66 (PA66) and poly(tetrafluoroethylene) (PTFE) used in this study was distributed by Quattroplast Ltd. (Budapest, Hungary) and produced by Ensinger GmbH (Nufringen, Germany). Table 1 shows selected characteristic properties of the polymers. For the adhesive tests, rectangular specimens with dimensions of 25.4 × 100 × 2 mm^3^ were cut from commercial grade extruded plates using a metal saw disc. A long strip were cut parallel to the longer edge of the plate in a width of 25.4 mm followed by slicing the strip into the final size. The surfaces used for adhesion tests were first polished with silicon carbide abrasive paper (grit number P1200 and 2000) under wet conditions, followed by dry polishing with felt sheet. Subsequently, the samples were rinsed with distilled water and ethanol. A standard steel grade S235 (Ferroglobus Ltd., Budapest, Hungary) was used as a counterface in the adhesion tests. The steel surfaces were also polished with SiC abrasive paper (grit numbers 400 and 600) to a mean surface roughness of Ra = 0.07 ± 0.02 µm. Finally, the surface was chemically cleaned from organic contaminants.

### 2.2. Plasma Treatment

A diffuse coplanar surface barrier discharge (DCSBD) (manufactured by Roplass s.r.o., Brno, Czech Republic) plasma system was employed for surface activation [23,24]. The plasma panel consists of two systems of parallel strip-like electrodes (with typical dimensions of: 1.5 mm wide, 0.5 mm thick, 1 mm strip to strip) embedded in aluminum oxide matrix. The ceramic layer between electrodes and the plasma has a thickness of typically 0.4 mm [25]. A sketch of the plasma panel is illustrated in [25]. The plasma was ignited with a high frequency (10–20 kHz), high voltage with peak-to-peak values of 20 kV. The elementary discharge comprises a diffuse surface discharge developing over the metal electrodes and a filamentary streamer discharge created between the electrodes giving its H shape. With increasing voltage and absorbed power as more and more elementary discharges are generated, visually homogenous plasma can be reached. The high voltage applied may lead to the heating of the dielectric surface and of the surrounding gas, too. Oil is circulated over the system to keep the system at the lowest possible temperature. It also allows to keep the gas temperature around 320 K. The DCSBD plasma system was operated at a power of 320 W, which provided a quasi-homogeneous diffuse plasma with air as process gas. 

In the plasma tests one side of the polymer samples is treated with DCSBD plasma for 30 s. A constant distance of 0.5 mm was maintained between the DCSBD surface and the polymer specimen. The latter was gently moved back and forth within a 1 cm period distance along the DCSBD surface so as to provide an even more homogeneous diffuse plasma contact.

### 2.3. Adhesive Testing

Adhesion was tested by lap-shear tests carried out on single lap joints of polymer/polymer or polymer/steel pairs with a bonded area of 25.4 mm × 12.5 mm according to DIN EN 1465. Four commercial adhesives were employed including a cyanoacrylate (Loctite 406), a 2-component epoxy adhesive (Loctite 9466) and two acrylic based adhesives (Loctite 330, 2-component glue: tetrahydrofurfuryl methacrylate, alkyl methacrylate, organoboron amine and Loctite 3035, 2-component glue designed for bonding low energy plastics without using primer) from the same manufacturer (Henkel AG & Co., Düsseldorf, Germany). The adhesives were applied onto the bonded area with a controlled thickness of 0.1 mm. According to manufacturer’s recommendation a primer (Loctite 770) was applied on the polymer surface prior to using the cyanoacrylate type adhesive. During curing a constant normal load of 5 N was maintained for 24 h over the bonded area. The plasma treated specimens were glued right after treatment and stored in a plastic box until testing. The pulling tests were performed using a universal mechanical tensile bench (Zwick Roell Z100, max. 100 kN Zwick GmbH & Co. KG, Ulm, Germany) with a pulling speed of 1.3 mm/min. The maximum load upon failure with respect to the bonded surface area was used to calculate the shear strength of the adhesive bond.

### 2.4. Surface Characterization

The chemical, morphological and energetic properties of the plasma treated surface were determined by X-ray photoelectron spectroscopy (XPS), sessile drop contact angle measurements (Advex Instruments, Brno, Czech Republic) and 3D non-contact optical profilometry (CCI Optics, Taylor-Hobson CCI HD 3D optical profiler, Leicester, UK). 

Apparent contact angle measurements were performed by the static sessile drop method at room temperature using double distilled water and diiodomethane (Sigma-Aldrich, Reagent Plus 99% grade, Budapest, Hungary), applying the SEE System apparatus (Advex Instruments, Brno, Czech Republic). The measuring liquids were deposited in 2 µL droplets by Hamilton syringe. The apparent contact angle values were presented as the average of five consecutive measurements, performed always on previously non-wetted parts of the samples. The surface free energy with its polar and dispersive components was calculated following the Owens-Wendt method [26]. 

A Kratos XSAM 800 spectrometer (Kratos Analytical Ltd., Manchester, UK) was used to record the X-ray photoelectron spectra. Mg Kα_1,2_ (1253.6 eV) excitation and fixed analyzer transmission (FAT) mode were used. Survey spectra were recorded in the 150–1300 eV kinetic energy range with 0.5 eV steps. Photoelectron lines of the constituent elements (C1s, O1s, N1s and F1s) were recorded by 0.1 eV steps. The C1s line (binding energy, BE = 285.0 eV) of the hydrocarbon type component of the polymers was used as a reference in the spectra. For peak decomposition a Gaussian-Lorenzian peak shape with 70/30 ratio was used. Quantitative analysis, based on peak area intensities after removal of the Shirley-type background, was performed by the Kratos Vision 2 and by the XPS MultiQuant programs [27], using experimentally determined photo-ionization cross-section data of Evans et al. [28] and asymmetry parameters of Reilman et al. [29]. Surface chemical compositions were calculated by the “infinitely thick sample” model. It assumes that all components are homogeneously distributed within the sampling depth detected by XPS. Measurements were performed on unique samples thus scatter of data cannot be supplied. The error of the quantitative XPS results, as generally accepted, is ±5 relative %.

The 3D surface characteristics were measured using the Taylor Hobson Coherence Correlation Interferometry CCI HD M112-4449-01 (CCI, Taylor Hobson, Leicester, UK) HD interferometer. A 50× magnification was used for the non-contact surface characterization where a field of view of 350 × 350 µm was studied at different location within the same sample. Different 3D surface parameters such as Sa (arithmetic mean height), Sz (maximum height), Sku (skewness) and Ssk (kurtosis) were extracted using the Talymap tool. Prior to the extraction of the 3D parameters the surface data was pre-processed by leveling for the horizontal plane and a threshold was set to a cut-off value. The uncertainty of the measurement is typically less than 5 relative percent. 

## 3. Results and Discussion

### 3.1. Contact Angle Measurements

**Figure 1:**
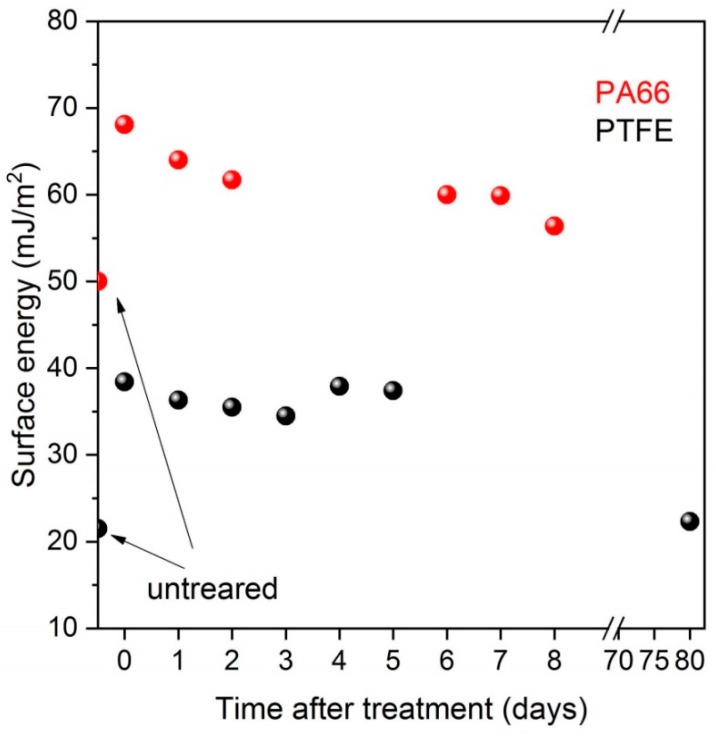
Surface energy values of the investigated polymers after DCSBD treatment as a function of time.

Table 2 shows the average apparent contact angle values of water and diiodomethane as well as the calculated surface energy values including both the polar and dispersive components for the pristine and DCSBD treated samples. While both investigated polymers are considered hydrophobic according to their high water contact angle values, PTFE shows an extremity among polymers with its 108° WCA value. Considerable decrease was reported in the apparent water contact angle values for a range of polymers on cold plasma treatment [30,31]. We also found that a 30 s DCSBD plasma treatment significantly decreased the apparent water contact angles from 63° to 33° for PA66, however, the overall decrease was negligible for PTFE reaching a 101° WCA. We examined the effect of plasma treatment up to 10 min for PTFE even though the efficacy of extended treatment time was doubted [29]. We found that prolonged treatment time, indeed, did not significantly improve the wettability of PA66. However, we also found that PTFE is an exception since the WCA value was gradually decreased over the treatment time to reach a final 68° after 10 min. Although, the overall decrease was significant, the PTFE remained hydrophobic that cannot be further improved by prolonged treatment. The plasma treatment time of the specimens for the adhesion and tribological tests was chosen as 30 and 300 s for PA66 and PTFE, respectively, considering the aforementioned WCA results. Parallel with the decrease of the apparent water contact angles the surface energies increased primarily due to the rise in the polar components. The effect of plasma treatment, however, is reported to vanish after longer period due to the surface reactivity and reorientation of polymer chains. We also found a gradually decreasing trend in the surface energy for both polymers in the subsequent days of the treatment (Figure 1). After 3 months of the treatment the surface energy of PTFE was completely restored to its original value.

### 3.2. Surface Analysis

We employed XPS analysis to monitor the changes in surface chemistry of the investigated polymers on plasma activation. Chemical compositions (atomic %) of the polymer surfaces before and after plasma treatment are presented in Table 3. High resolution spectra of the C1s and O1s lines were used to determine quality and quantity of the developed polar groups on the surface. The assignment of the peak components to chemical features is also summarized in Table 4.

**Figure 2:**
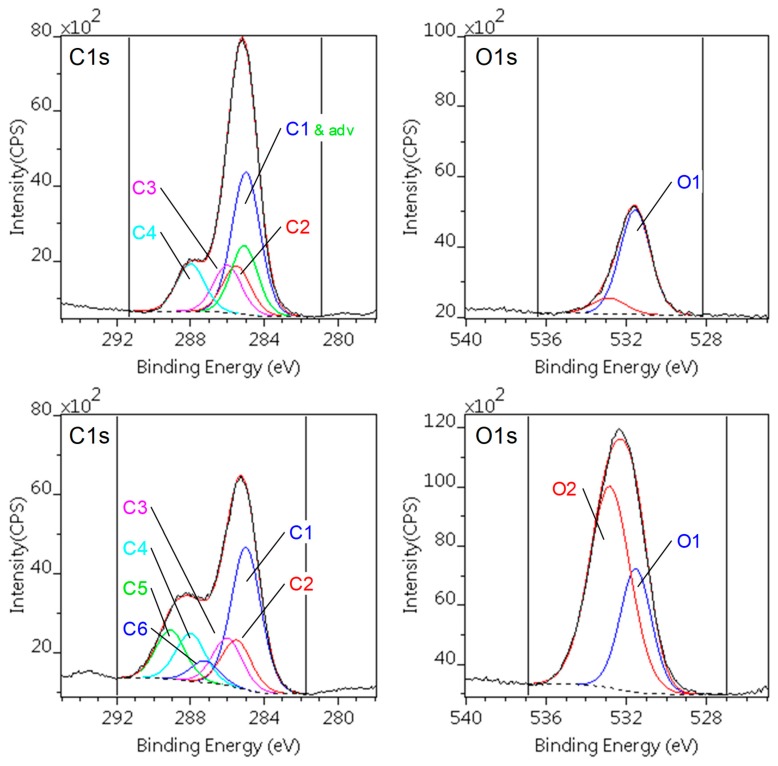
C1s and O1s photoelectron spectra of the pristine (upper) and DCSBD treated (lower) PA66 sample. On the untreated sample the C1 component coincides with the adventitious carbon.

The decomposed C1s and O1s spectra of the PA66 samples are shown in Figure 2. The surface of the pristine PA66 sample, despite of a small amount of adventitious carbon and extra oxygen, was almost equivalent to those described in the literature [32]. After plasma treatment the carbonaceous contamination removed and oxygen content is significantly increased, as shown Figure 2 (lower panes) and in Table 4. The ratio of the PA66 carbon species are unchanged but two additional components (C5, C6) appeared, corresponding to carboxylic (acid, ester) environment. The increment of the oxygen (O_2_) can also be assigned to carboxylic groups. The concentration of nitrogen is slightly increased.

The increased amount of polar groups incorporated onto the surface layer explicates the increased wettability. The obtained results are in agreement with findings of earlier papers, [19,33] although surface modification was carried out by other types of non-equilibrium plasma method.

**Figure 3:**
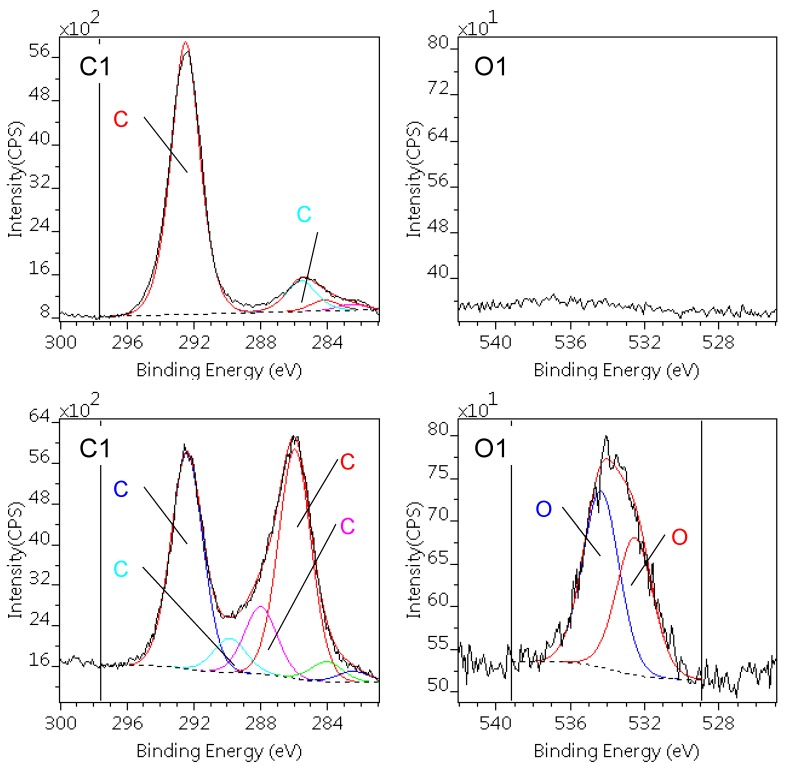
C1s and O1s photoelectron spectra of the pristine (upper) and DCSBD treated (lower) PTFE sample (unmarked C1s components are X-ray satellites).

The similarly decomposed C1s and O1s spectra of the PTFE samples are depicted in Figure 3. The surface of the pristine PTFE sample is notably clean: beside a low level of hydrocarbon type contamination no oxygen could be detected. The peak positions and intensity are in full agreement with the literature data [30].

The plasma treatment caused a significant defluoronation in the surface layer indicated by the F/C atomic ratio. In parallel, new components appeared in the C1s spectral region, corresponding to the various C–F species, enumerated in Table 4 and illustrated in the lower panes of Figure 3. Only a small amount of oxygen and nitrogen were incorporated into the surface, indicating low level of surface oxidation. The related carbon partner of these atoms, like C–OH, C–N, O–C–O, cannot be separated from the Fluor containing species (C1, C2).

The better wettability of DCSBD treated PTFE can be ascribed to these polar groups but their population was not sufficient to make the surface hydrophilic. Other studies confirm that surface modification of PTFE is not effective using air plasma, while addition of H_2_ even in small amount greatly improves the wettability [34]. 

### 3.3. Surface Topography

To monitor the surface topography of pristine and plasma-treated samples we used 3D surface scans by non-contact profilometry. Surface characteristics is one of the critical parameter which may contribute to a tribo-system hence, the same was evaluated for the treated and untreated specimen of PA66 and PTFE. To fully understand the effects of irradiation on the surface characteristics, a systematic approach was followed. Both PTFE and PA66 were machined and subsequently polished with an established methodology for achieving a uniform surface roughness. However the material removal during the polishing operation does rely on the material properties. Hence the same procedure on both the materials (PA66 and PTFE) tends to have different roughness values. However, within the same specimen the roughness remains the same between the untreated samples. Surface investigation was carried out for both grades for untreated and treated samples. In regards to the treated samples the influence of idle tie after treatment process were investigated. An interval of 24 h and 800 h were used to study the change in surface growth as a consequence of idle time. Table 5. clearly shows the 3D surface characteristics of the untreated and the treated PA66 and PTFE on the basis of a measurement performed on a representative field of 350 × 350 µm. 

In regards to the surface characteristics the effect of irradiation is significant for both the PA66 and the PTFE. In case of PTFE the roughness remains the same after 24 h idle time, however after 800 h the roughness Sa increases by 35%. The Sz which represents the maximum peak to valley distance increases by 69% between the untreated and the treated (after 800 h) for the PTFE. However, there is a difference while considering the idle time between the two materials However, the idle time plays a major role in the case of PTFE where sufficient idle time (800 h) is required for altering the surface roughness.

**Figure 4:**
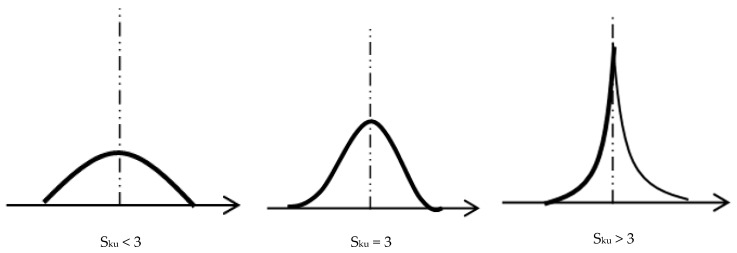
Probability density functions for random distributions with different Kurtosis.

**Figure 5:**
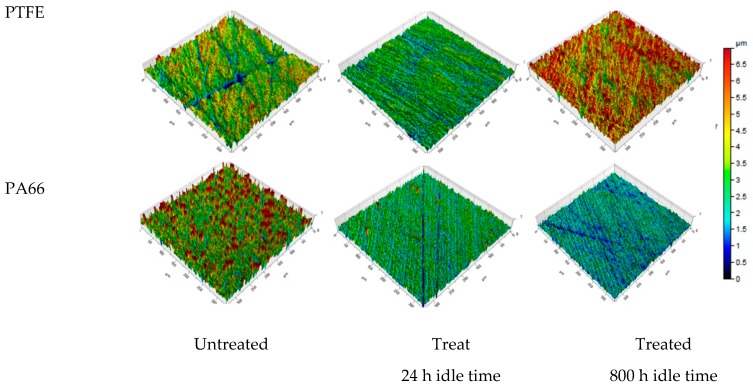
3D topography of treated and untreated specimen for PA66 and PTFE grades.

Considering the initial roughness, both PTFE and PA66 has significant roughness from the polishing methodology. PA66 has higher roughness in comparison to the PTFE specimen. This difference can be attributed to the material response on the polishing procedure. Based on the skewness a positive value for PA66 and a negative value for PTFE with 1.89 Ssk and −0.78 Ssk were found respectively. Hence the distribution of peaks is dominant in the case of PA66 and the PTFE surface was dominated by valleys. However, the Kurtosis values were found to be above 3 in both PTFE and PA66 specimen surface. This means the morphology in both cases points out sharp spikes as a consequence of polishing (Figure 4). 3D surface topography for both treated and untreated specimens of PA66 and PTFE is presented in Figure 5. It is noteworthy to mention that all the 3D images were normalized to the same scale and hence the color code represents the degree of roughness. The red color peaks in the 3D roughness profile of untreated PA66 (Figure 5) clearly indicates the increased roughness when compared with the untreated PTFE. In the untreated specimen for both PTFE and PA66, the surface roughness does not change even after 800 h of idle time. 

Considering the difference in roughness characteristics between untreated and treated specimen, significant difference in surface characteristics were found between PA66 and PTFE. A clear evidence of diminishing peaks was found in case of PA66. This is justified by the change in skewness from a positive to a negative value. This means the roughness peak from an individual asperity has melted as a consequence of irradiation. Such a melt stage loses its strength to sustain its structure (increased peak to valley distance). On cooling the melt phase solidifies within the axis of individual asperity, however the width of the asperity may increase from the settling of the molten phase and thus the height loss at an asperity scale. The negative skewness after 800 h of idle time for the treated PA66 specimen clear represents the surface with dominated valleys. The curvature of the peak in the asperity changes and hence represents a profile with a negative skewness. 

In regards to the PTFE the skewness does not change significantly, hence the morphology remains the same. However, the peak to valley distance (Sz) and the 3D roughness Sa changes significantly by 69% and 36%, respectively. This means the peaks have formed in the course of idle time (800 h) for the irradiated specimen. During the treatment process the O_2_ and N_2_ in the ambience may have introduced a chemical change and hence the polar groups were internally stressed to form rough surface. It is evident from the surface characterization that the treatment process will certainly affect the surface characteristics in one way or the other with respect to the polymer group. Knowing that the surface characteristics are affected one may expect a difference in the tribological nature between PA66 and PTFE for treated and untreated specimen. 

### 3.4. Adhesive Tests

**Figure 6:**
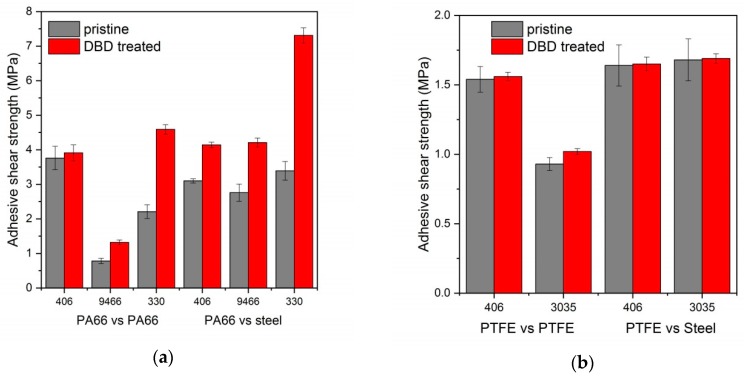
Adhesive shear strength of pristine (grey) and plasma-treated (red) polymer/polymer and polymer/steel joints using various adhesives for PA66 (**a**) and PTFE (**b**).

Figure 6a,b show the shear strength of pristine and DCSBD treated polymer/polymer and polymer/steel joints evaluated by lap-shear tests for PA66 and PTFE, respectively. Applying the adhesives on the untreated PA66 surfaces the obtained shear strength showed great variation. Adhesion was three times higher using a cyanoacrylate-based glue (Loctite 406) as compared to an epoxy glue (Loctite 9466). For polymer/steel joints the adhesion strength exhibited only minor variance among the applied types of adhesive. After setting all the adhesives became rigid and peeled off the surface of the PA66 polymer that is capable of significant strain. On DCSBD treatment the adhesion strength improved more or less for each types of adhesive. The highest improvement both for the polymer/polymer and polymer/steel joints occurred when a two component acrylic adhesive (Loctite 330) was employed. It seems obvious that the enhancement of the adhesion can be attributed to the higher wettability and surface polarity of the DCSBD treated polymer surfaces. In addition of the increased adhesion strength, the reliability of the joints also improved as it was indicated by the decrease of the statistical deviation of the shear strength (5 repetitions) after plasma treatment for all adhesives and joint types from about 9% for the pristine samples to 4% in average for the plasma-treated PA66. 

The adhesion of pristine PTFE is rather poor for any type of adhesive and joints relation. The adhesives detach from the surface easily implying lack of strong bonds between the polymer surface and the adhesive. On plasma treatment the adhesion did not change for any adhesives and for either polymer/polymer or polymer/steel joints. Even though, one beneficial effect of plasma treatment for the adhesion can be revealed on studying the standard deviation of the adhesion strength. It significantly decreased after plasma treatment from 9% to 2–3% similarly to PA66. Comparing the sharply different adhesion behavior of the investigated two polymers on DCSBD treatment we can find only one crucial reason for the better performance of PA66. Plasma treatment created a lot more oxygen containing polar moieties on the surface of PA66 as compared to PTFE. The higher polarity of the surface increased the surface energy of PA66 by ca.30 mJ/m^2^ above that of PTFE. 

Failures observed with respect to the different joints and polymers could mainly be ascribed to adhesive failure on the surface of the polymer or steel, while cohesive failure within the adhesive film or cracking in the bulk polymer could not be detected. For the pristine PA66 the adhesives detached from the polymer surface, whereas after DCSBD treatment for polymer/steel joint relations the adhesives rather peeled off from the steel surface indicating such a stronger bond to the polymer. For pristine PTFE adhesive failure occurred primarily on the polymer surface that did not changed after treatment but failure occurred at a more or less, higher applied shear stress. 

### 3.5. Tribology

**Figure 7:**
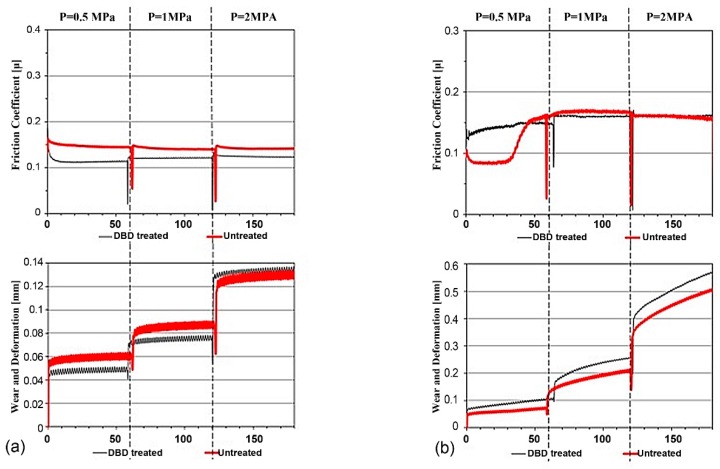
Coefficient of friction and wear loss for (**a**) PA66 and (**b**) PTFE.

Tribological tests were performed for both treated and untreated specimen using a pin on disc tester under lubricated condition. The friction and wear response of both treated and untreated specimens of PA66 and PTFE were show in Figure 7a,b. It is evident from the figure that significant differences in tribological properties were observed between treated and untreated specimen for PA66 and PTFE specimen. In regards to the friction characteristics a clear running in phase with a transition to steady state was found for both materials (except for PTFE tested at 0.5 MPa contact pressure). For PA66 the coefficient of friction in case of treated specimen was found to be lower than the untreated in all loading conditions. However, the PTFE specimens show similar friction trend between treated and untreated specimen for 1 MPa and 2 MPa contact pressure. The decrease in friction characteristics in PA66 between the treated and the untreated is a consequence of a decrease in surface roughness (Sa decreases by 60%) where the smoothening increases the adhesion of lubricant to the contact surface. Additionally, the morphology with reduced peaks and increase base of individual asperity increases the adhesion characteristics. In case of PTFE the roughness vaguely decreases and the morphology remains the same and hence the difference in coefficient of friction between the treated and the untreated is rather limited. The asperity between the PTFE and the counterface increases as the consequence of increase in roughness. Hence one may conclude that the treatment in the PA66 favors low friction characteristics. 

In regards to the wear characteristics an inverse effects in wear properties were observed between the two materials (PA66 and PTFE) for treated and untreated specimen. Though PA66 is meant to be more wear resistant than the PTFE a comparison based on the relative wear between the treated and the untreated specimen will explicate the influence of treatment process on the wear resistance. In case of the PA66 the treatment process improves the wear resistance in all three load cases. The effects are significant for low and medium load condition. In case of the PTFE specimen wear resistance decreases as a consequence of treatment process. This can be attributed to the increase in roughness, which may initiate the contact between asperities of the test material and the counterface. Such an interaction may also increase the coefficient of friction. 

## 4. Conclusions

The atmospheric air dielectric coplanar surface barrier discharge (DCSBD) plasma treatment induced various morphological and chemical alterations on the topmost surface of the studied engineering polymers (PA66 and PTFE). For plasma treatment significant amount of new oxygen containing moieties formed on the surface of PA66 according to XPS analysis. The higher number of the polar groups on the surface increased the surface energy and improved the wettability. Adhesion properties of PA66 were also improved for all adhesives both for polymer/polymer and polymer/steel joints. However, outstanding improvement could be observed only for the acrylic type (Loctite 330) of adhesives. DSCBD treated PA66 exhibited slightly enhanced tribological properties, too, with regards to friction coefficient and wear.

Although, the plasma treatment also affected the PTFE and the F to C atomic ratio greatly decreased, the surface was only slightly oxidized. Albeit the surface energy almost doubled, the surface remained still hydrophobic. Considerable changes neither in the adhesion nor in the tribological properties could be observed. However, a positive effect of the plasma treatment for PTFE that the reliability of the adhesive joints greatly improved that was apparent from the considerably smaller standard deviation of the adhesive forces.

## Figures and Tables

**Table 1:** Major properties of the studied polymers.

Property	PA66	PTFE
Density (g·cm^−3^)	1.15	2.16
Tensile strength (MPa)	85	22
Elasticity modulus MPa)	1850	400–550
Shore D hardness	78–88	55
Thermal conductivity (W·m^−1^·K^−1^)	0.25–0.35	0.25–0.30
Melting temperature (°C)	260	-

**Table 2:** Contact angle and surface energy values of the pristine and the plasma treated polymer samples measured at various times after treatment.

Sample	θ_w_ (°)	θ_CH2l2_ (°)	γ_pol_ (mJ/m^2^)	γ_disp_ (mJ/m^2^)	γ_tot_ (mJ/m^2^)
PA6.6 pristine	63 ± 2.3	42 ± 2.5	11.2	38.7	50.0 ± 2.4
PA6.6 treated, 30 s	33 ± 2.6	27 ± 2.7	24.0	45.5	69.5 ± 4.9
PA6.6 treated, 60 s	29 ± 3.6	27 ± 1.2	25.7	45.7	71.4 ± 4.8
PA6.6 treated, 180 s	30 ± 2.6	24 ± 2.5	25.1	46.7	71.8 ± 5.4
PTFE pristine	108 ± 1.5	73 ± 3.2	0.2	21.2	21.5 ± 0.5
PTFE treated, 30 s	101 ± 3.4	73 ± 4.6	1.2	21.1	22.4 ± 0.7
PTFE treated, 60 s	105 ± 2.5	62 ± 3.9	0.1	27.6	27.7 ± 0.7
PTFE treated, 180 s	75 ± 1.0	56 ± 1.0	7.5	30.9	38.4 ± 0.2
PTFE treated, 300 s	68 ± 3.7	61 ± 1.6	12.4	28.2	40.6 ± 0.5
PTFE treated, 600 s	65 ± 1.5	60 ± 2.4	13.8	28.5	42.3 ± 0.4

**Table 3:** Surface composition (atomic %) of the pristine and plasma treated polymer samples determined by XPS (the error of the quantitative results is ±5 rel.%).

	Chemical Composition, %				Atomic Ratio		
Sample	C	O	N	F	O/C	N/C	F/C
PA66 pristine	76.1	12.0	11.9	-	0.16	0.15	-
PA66 treated	58.5	27.8	13.7	-	0.47	0.23	-
PTFE pristine	33.5	-	-	66.5	0	0	1.98
PTFE treated	49.5	3.4	2.1	45.0	0.07	0.04	0.91

**Table 4:** Surface composition and chemical state assignments of the C1s components of the pristine and plasma treated PA66 and PTFE polymer samples.

C Components	Composition (Atomic %)		Binding Energy (eV)	Chemical States
	Pristine	Plasma Treated		
PA66				
C1	38.0	23.8	285.0 ± 0.1	C–C, C–H
C2	12.7	7.9	285.3 ± 0.1	CH–C=O
C3	12.7	7.9	286.0 ± 0.1	*C–N–C=O
C4	12.7	7.9	288.0 ± 0.1	C–N–*C=O
C5		7.7	289.0 ± 0.2	O=*C–O(H)
C6		3.3	287.0 ± 0.2	*C–O–C=O, epoxy
PTFE				
C1	0	21.0	286.4 ± 0.1	–*CH2–CF2 C–O–C, C–OH, C–N
C2	0	6.1	287.9 ± 0.1	–CH2–*CHF– C=O, O–C–O, N–C–O
C3	0	3.1	289.8 ± 0.1	*CF(CF3) –CF2– –CF2–CH2–
CF	33.5	19.3	292.5 ± 0.1	–CF2–CF2–

**Table 5:** 3D surface characteristics of PTFE and PA66.

Test Materials	PTFE				PA66			
3D Parameters	Sa	Sz	Sku	Ssk	Sa	Sz	Sku	Ssk
Untreated	0.56	5.4	3.99	−0.78	0.91	14.7	10.9	1.89
Treated 24 h	0.5	4.29	3.56	−0.6	0.44	7.06	6.27	0.03
Treated 800 h	0.76	9.12	4.47	−0.7	0.36	4.84	4.12	−0.01
